# Effects of Different Exercise Training Protocols on Gene Expression of Rac1 and PAK1 in Healthy Rat Fast- and Slow-Type Muscles

**DOI:** 10.3389/fphys.2020.584661

**Published:** 2020-11-19

**Authors:** Saara Laine, Heidi Högel, Tamiko Ishizu, Jussi Toivanen, Minna Yli-Karjanmaa, Tove J. Grönroos, Juha Rantala, Rami Mäkelä, Jarna C. Hannukainen, Kari K. Kalliokoski, Ilkka Heinonen

**Affiliations:** ^1^Turku PET Centre, Turku University Hospital, University of Turku, Turku, Finland; ^2^MediCity Research Laboratory, University of Turku, Turku, Finland; ^3^Turku Centre for Biotechnology, University of Turku, Åbo Akademi University, Turku, Finland; ^4^Natural Resources Institute Finland (Luke), Jokioinen, Finland; ^5^Institute of Biomedicine, University of Turku, Turku, Finland; ^6^TuDMM Doctoral Programmes, University of Turku, Turku, Finland; ^7^Misvik Biology, Turku, Finland; ^8^Rydberg Laboratory of Applied Sciences, University of Halmstad, Halmstad, Sweden

**Keywords:** skeletal muscle, exercise, glucose uptake, HIIT, MICT, Rac1, PAK1, RPPA

## Abstract

**Purpose:**

Rac1 and its downstream target PAK1 are novel regulators of insulin and exercise-induced glucose uptake in skeletal muscle. However, it is not yet understood how different training intensities affect the expression of these proteins. Therefore, we studied the effects of *high-intensity interval training* (HIIT) and *moderate-intensity continuous training* (MICT) on Rac1 and PAK1 expression in fast-type (*gastrocnemius*, GC) and slow-type (*soleus*, SOL) muscles in rats after HIIT and MICT swimming exercises.

**Methods:**

The mRNA expression was determined using qPCR and protein expression levels with reverse-phase protein microarray (RPPA).

**Results:**

HIIT significantly *decreased Rac1* mRNA expression in GC compared to MICT (*p* = 0.003) and to the control group (CON) (*p* = 0.001). At the protein level Rac1 was *increased* in GC in both training groups, but only the difference between HIIT and CON was significant (*p* = 0.02). HIIT caused significant *decrease* of *PAK1* mRNA expression in GC compared to MICT (*p* = 0.007) and to CON (*p* = 0.001). At the protein level, HIIT increased PAK1 expression in GC compared to MICT and CON (by ∼17%), but the difference was not statistically significant (*p* = 0.3, *p* = 0.2, respectively). There were no significant differences in the Rac1 or PAK1 expression in SOL between the groups.

**Conclusion:**

Our results indicate that HIIT, but not MICT, *decreases* Rac1 and PAK1 mRNA expression and *increases* the protein expression of especially Rac1 but only in fast-type muscle. These exercise training findings may reveal new therapeutic targets to treat patients with metabolic diseases.

## Introduction

Muscle contractions increase glucose uptake (GU) via GLUT4 (*glucose transporter type 4*) mediated mechanisms (Sahlin, 1990; Warburton et al., 2006). Specific signaling pathways remain, however, incompletely elucidated but recent studies indicate that in addition to insulin-stimulated GU, Rac1/PAK1 signaling seems to play an important role also in contraction-mediated GU (Sylow et al., 2013b, 2016).

Ras-related C3 botulinum toxin substrate 1 (Rac1) is a small (∼21 kDa) signaling protein belonging to the Rac subfamily of the Rho family of GTPases, which are responsible for many cell functions such as cell growth, remodeling of the cytoskeleton, and activation of protein kinases. GTPases of cell membrane alternate between active guanosine triphosphate (GTP) and inactive guanosine diphosphate (GPD) form. GTP binds to different effector proteins and regulates multiple cell reactions.

p21 protein (Cdc42/Rac)-activated kinase 1 (PAK1) is a serine/threonine-protein kinase and a Rac1 target. GTP-bound activated Rac1 binds to PAK1 resulting in conformational change and autophosphorylation on threonine 423 (Thr423) of PAK1 (Chong et al., 2001). The consecutive cytoskeleton remodeling and translocation of GLUT4 to the cell membrane increases GU into the muscle (Tunduguru et al., 2017). Recent data suggest that Rac1 is required for skeletal muscle GU in both insulin- and exercise-mediated signaling (Sylow et al., 2013a,b). Disturbances in the function of Rac1 signaling have been observed in insulin resistance in both human and mice (Sylow et al., 2013a), but the effects of different exercise training intensities on the expression of PAK1 and Rac1 remain sparsely characterized.

Thus, the aim of this study was to compare the effects of high-intensity interval training (HIIT) and moderate-intensity continuous training (MICT) on Rac1 and PAK1 gene and protein expression in fast-type *gastrocnemius* (GC) and slow-type *soleus* (SOL) muscles in healthy male rats. It was hypothesized that HIIT increases the expression of Rac1 and PAK1 in both muscle types, while MICT did so only in SOL.

## Materials and Methods

### Animals

Twenty-three healthy male Wistar rats (Charles River Laboratories, Germany) 8–12 weeks of age were randomly divided into three groups: HIIT *n* = 8, MICT *n* = 7, and control (CON) *n* = 8 [mean weights at the start of the exercise intervention: 295 g (*SD* = 18.9 g), 286 g (*SD* = 14.1 g), 282 g (*SD* = 15.6 g), respectively]. Animals were housed under standard conditions (temperature 21°C, humidity 55 ± 5%, 12:12 light-dark cycle) with food and water available *ad libitum*. The National Animal Experiment Board (ESAVI/5053/04.10.03/2011) approved all animal procedures, and all studies were performed in accordance with the guidelines of the European Community Council Directives 86/609/EEC.

### Exercise Interventions and Sampling

Before and after the interventions, the aerobic capacity of the animals was studied by measuring the maximal oxygen consumption (VO_2max_) with rat single-lane treadmill (Panlab-Harvard Apparatus). Animals were accustomed to the treadmill for 3 days before the VO_2max_ test. The test started after a warm-up period. During the test, the angle of the treadmill was 25°, and the speed was increased by 3 cm/s after every other minute until exhaustion.

Animals performed 10 exercise sessions within 2 weeks. Each HIIT session consisted of eight to ten 30 s swimming bouts with 1-min resting period after each bout. HIIT group animals wore weight vests of 30–50 g. Vest weight was determined for all individuals separately on each exercise session and bout to acquire as maximal effort as possible. MICT group animals swam without any additional weights and started with 40-min continuous swimming exercise, after which the duration was increased by 10 min every second session until there were two 80-min sessions. Animals were sacrificed 5 days after the last exercise session [mean weights: HIIT, 367 g (*SD* = 15.5 g); MICT, 374 g (*SD* = 10.02 g); C, 367 g (*SD* = 22.2 g)]. Calf muscles GC and SOL were collected and snap frozen with dry ice in isopentane and stored at −70°C.

### Gene Expression Analysis

The frozen muscle samples were homogenized in lysis buffer (Nucleospin^®^ RNA lysis buffer, Macherey-Nagel) with an Ultra Turrax T25 (Janke & Kankel IKA^®^ Labotechnik) on ice. The RNA was extracted using an RNA extraction kit (Nucleospin^®^ RNA XS—isolation of RNA from fibrous tissue, rw 01, Macherey-Nagel) according to the manufacturer’s protocol. The RNA concentration and purity of the samples were measured using Nanodrop (Thermo Fisher Scientific). The reverse transcription was performed using M-MuLV RNase H-reverse transcriptase (Promega) according to the manufacturer’s protocol. For gene expression analysis, PrimeTime^®^ qPCR Probe Assays (IDT) for RAC1, PAK1, and β-actin (ACTB) were used. Reactions were run using QuantStudio 12K Flex Real-Time PCR system (qPCR, Thermo Fisher Scientific) at the Finnish Functional Genomics Centre at Turku Centre for Biotechnology. Gene expression was normalized using ACTB as a housekeeping gene, and the expression was confirmed to be the same across the two muscles and the interventions. ΔΔCt method {2 ^–(ΔΔ*Ct)*^ [ΔCt = Ct_*mean*_ (gene of interest) – Ct_*mean*_ (ACTB); ΔΔCt = ΔCt (sample) – ΔCt_*mean*_(control)]} (Livak and Schmittgen, 2001) was used to quantify mRNA levels.

### Protein Expression Analysis

The protein expression was studied using validated reverse-phase protein microarray method (RPPA) (Spurrier et al., 2008; Leivonen et al., 2009). Briefly, the muscle samples were homogenized (100 mM Tris; pH 8; 0.5% SDS; 10 mM DTT; 50 mM NaF; 5 mM Na_3_VO_4_) with pestle (Scienceware^®^ Pestles and tubes), denatured in a water bath and printed onto a microscope glass (ONCYTE AVID NC16; Grace Bio-Labs Inc.) (20°C, humidity 64%) by Microarray robot (QArray Mini, Genetix). Arrays were stained (SYPRO^®^ Ruby Protein blot stain, S-11791) and detected with a laser scanner (LS Reloaded, Tecan).

For protein detection screened antibodies (a detailed description about the screening process is provided in the Supplementary Material) against Rac1 (ab33186, Abcam), phospho-PAK1 (Thr423) (PA5-12844, Thermo Fisher Scientific), and PAK1 (PA5-18557, Thermo Fisher Scientific) were used at dilution ratio 1:100 and 1:200, respectively. Antibodies were diluted in Odyssey Blocking Buffer (1xTBS 1:1; 0.5% Tween). Secondary antibodies anti-mouse (800CW Donkey anti-Mouse IgG), anti-goat (680RD Donkey anti-Goat IgG) (IRDye^®^, LI-COR Biosciences) and anti-rabbit (Goat anti-Rabbit IgG (H+L), Alexa Fluor 790, Thermo Fisher Scientific) were detected using the LI-COR Odyssey Imager (LI-COR Biosciences). The intensities were normalized to total protein expression using Array-Pro Analyzer microarray analysis software (Median Cybernetics Inc.). Normalization was done by dividing the net signal intensity of the antibody (raw signal – background = net signal) by the protein signal intensity (SYPRO^®^ Ruby Protein blot stain, S-11791) measured from the same sample.

### Statistical Analysis

For statistical analyses one-way ANOVA and Welch’s test was used to compare the gene expression between the groups and muscles. Two-tailed *t* test was used to compare the baseline differences between GC and SOL in CON group. Linear mixed model was used to compare the differences in maximal oxygen consumption between the groups. Multiple comparisons were made with Tukey honestly significant difference (HSD) test and Wilcoxon test. To achieve normal distribution of the data, logarithmic (log10) or square root transformations were performed when necessary. Results are shown as mean and standard deviation (SD). The significance level was set at 0.05. Analyses were performed using JMP Pro (JMP^®^, Version 13. SAS Institute Inc., Cary, NC, 1989-2019).

## Results

### Maximal Oxygen Consumption Was Improved in Both Training Groups

Maximal oxygen consumption (VO_2max_) was significantly improved in both HIIT and MICT group when compared to untrained CON group [Pre: HIIT, 73.63 (*SD* = 3.70); MICT, 71.51 (*SD* = 2.29); CON, 67.14 (*SD* = 4.19) ml min^–1^ kg^–0.75^; Post: HIIT, 76.12 (*SD* = 4.21); MICT, 74.20 (*SD* = 5.15); CON, 69.93 (*SD* = 4.28) ml min^–1^ kg^–0.75^, *p* = 0.0007, *p* = 0.027, respectively].

### HIIT Induces Rac1 Expression in Fast-Type Gastrocnemius Muscles

**FIGURE 1:**
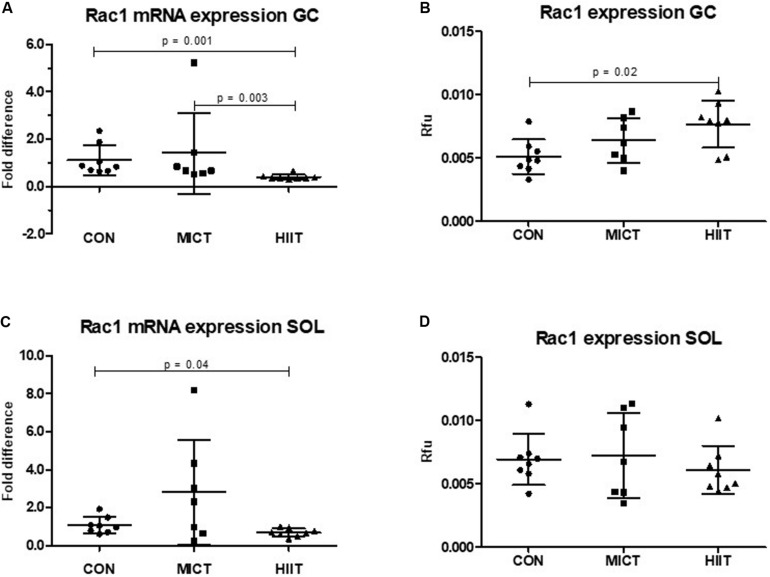
Comparison of the mRNA and protein expressions in HIIT and MICT groups vs. controls (CON). The effect of HIIT and MICT exercises on Rac1 mRNA expression **(A,C)** and Rac1 protein expression **(B,D)** in fast glycolytic GC (gastrocnemius) and slow oxidative SOL (soleus) muscles. The relative mRNA levels (fold difference) were calculated using ΔΔCt method normalized to β-actin (ACTB) reference gene. Protein level normalization was done by dividing the net signal intensity of the antibody (raw signal – background = net signal) by the protein signal intensity measured from the same sample. Rfu, relative fluorescence units; HIIT, high-intensity interval training; MICT, moderate-intensity continuous training.

Ras-related C3 botulinum toxin substrate 1 mRNA and protein expression level were higher in SOL compared to GC in untrained CON group (12% *p* = 0.62, 36% *p* = 0.05, respectively) (data not shown). HIIT group showed significantly lower *Rac1* mRNA expression in GC compared to MICT (*p* = 0.003) or to CON (*p* = 0.001) (Figure 1A). The similar trend was also seen in SOL, but the difference was only seen between HIIT and CON (*p* = 0.04) (Figure 1C). The *Rac1* mRNA expression in MICT group did not differ significantly in either muscle type compared to CON (Figures 1A,C). At protein expression level there was an increase of Rac1 in MICT and HIIT groups in GC compared to CON, although the only difference between HIIT and CON was statistically significant (*p* = 0.02) (Figure 1B). There were no significant differences in Rac1 protein levels in SOL between training groups and CON (Figure 1D).

### HIIT Activates PAK1 Expression in Fast-Type Gastrocnemius Muscles

**FIGURE 2:**
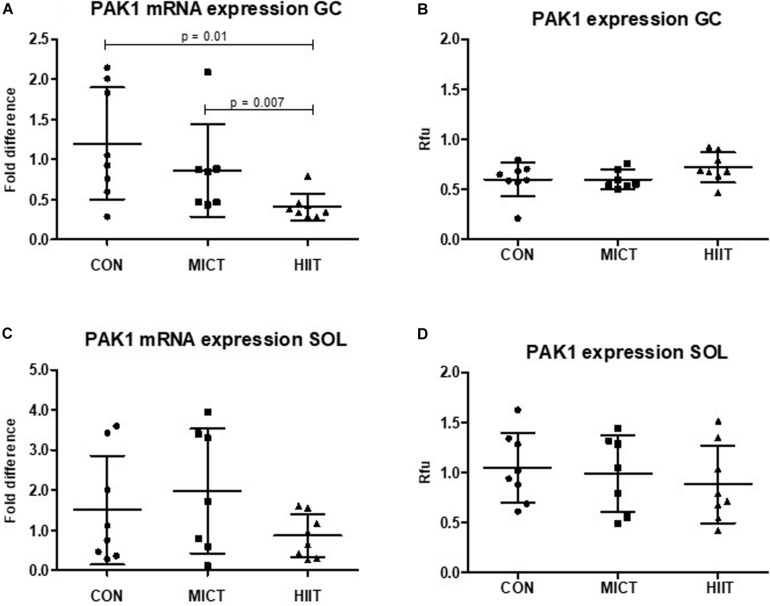
Comparison of the mRNA and protein expressions in HIIT and MICT groups vs. controls (CON). The effect of HIIT and MICT exercises on PAK1 mRNA expression **(A,C)** and PAK1 protein expression **(B,D)** in fast glycolytic GC (gastrocnemius) and slow oxidative SOL (soleus) muscles. The relative mRNA levels (fold difference) were calculated using ΔΔCt method normalized to β-actin (ACTB) reference gene. Protein level normalization was done by dividing the net signal intensity of the antibody (raw signal – background = net signal) by the protein signal intensity measured from the same sample. Rfu, relative fluorescence units; HIIT, high-intensity interval training; MICT, moderate-intensity continuous training.

**FIGURE 3:**
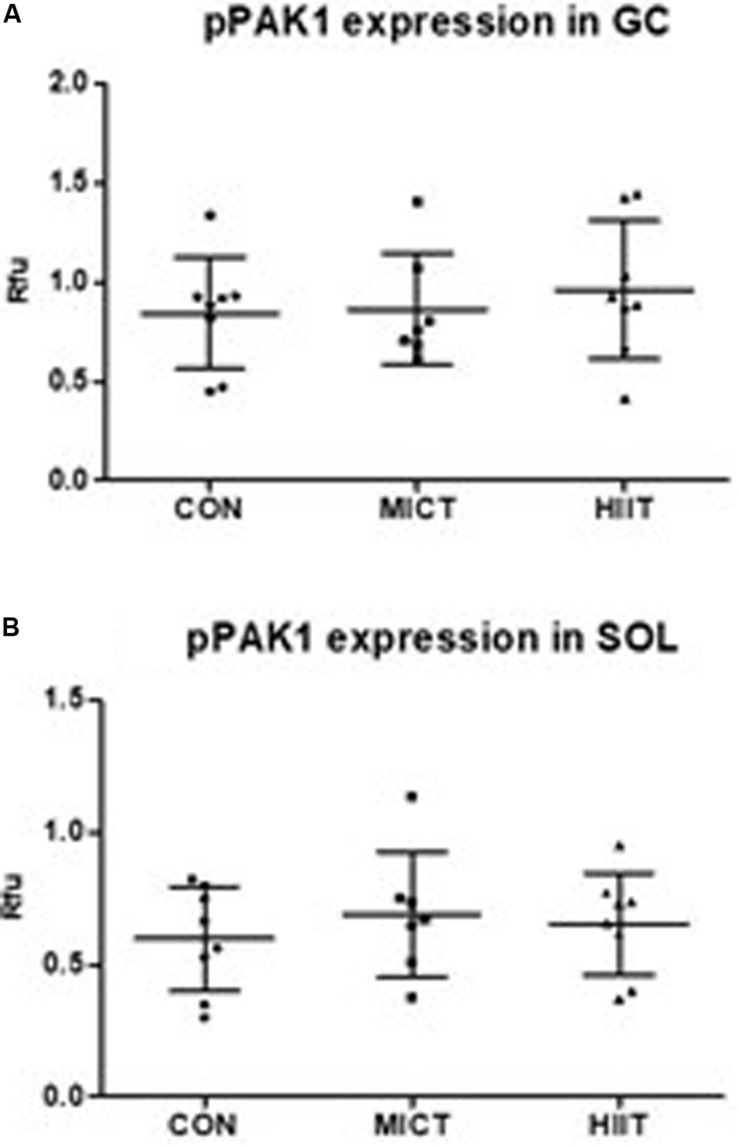
Comparison of protein expressions in HIIT and MICT groups vs. controls (CON). The effect of HIIT and MICT exercises on pPAK1 [phospho-PAK1 (Thr423)] expression **(A,B)** in fast glycolytic GC (gastrocnemius) and slow oxidative SOL (soleus) muscles. Protein level normalization was done by dividing the net signal intensity of the antibody (raw signal – background = net signal) by the protein signal intensity measured from the same sample. Rfu, relative fluorescence units; HIIT, high-intensity interval training; MICT, moderate-intensity continuous training.

p21 protein (Cdc42/Rac)-activated kinase 1 mRNA and protein expression level were higher in SOL compared to GC in untrained CON group (23% *p* = 0.54, 83% *p* = 0.006, respectively) (data not shown). A decrease in *PAK1* mRNA level was observed in GC in response to exercise, but only HIIT caused statistically significant decrease compared to MICT (*p* = 0.007) or to CON (*p* = 0.01) (Figure 2A). In SOL there were no differences in *PAK1* mRNA expression between groups (Figure 2C). At protein expression level, HIIT showed higher PAK1 and phospho-PAK1 expression in GC compared to MICT group and to the CON group, but the difference was not statistically significant (*p* > 0.2 in all comparisons) (Figures 2B, 3A). There were no differences in PAK1 or phospho-PAK1 expression between groups in SOL (Figures 2D, 3B).

## Discussion

Exercise and insulin cause Rac1 and PAK1 activation in both human and in mice skeletal muscle (Sylow et al., 2013a). Moreover, exercise intensity has an impact on glucose metabolism as HIIT might provide the same or even better results than MICT (Babraj et al., 2009; Eskelinen et al., 2015; Sjoros et al., 2017). Rac1 and PAK1 are proposed regulators of insulin signaling and GU in skeletal muscle. Especially the role of Rac1 signaling has been studied intensively (Sylow et al., 2013a,b, 2014a,b, 2016), but changes in expression levels of both mRNA and protein and effects of different exercise training intensities have not been fully considered previously.

In our study only HIIT had a significant impact on Rac1 and PAK1 mRNA and Rac1 protein expression levels, and change occurred only in GC. Mammalian GC consists mainly of fast IIa, IIx, and IIb fibers (Schiaffino and Reggiani, 2011), and it is known that HIIT is required to recruit these fibers (Armstrong and Laughlin, 1985; Laughlin et al., 1988; Kohn et al., 2011). Thus, our results suggest that muscle fiber recruitment pattern accounts for the findings in our study. The evidence of changes in muscle glucose metabolism in response to HIIT is not consistent (Jelleyman et al., 2015). However, our results indicate intensity- and muscle type-specific changes in Rac1 and PAK1, suggesting possible changes in GU in these muscle types. This may be connected to muscle-specific GU, which is reflected by decreased GU heterogeneity with increasing exercise intensities in skeletal muscles (Heinonen et al., 2012).

The activation of PAK1 by Rac1-GTP increases in an intensity-dependent manner in SOL in response to acute exercise (Sylow et al., 2013b). We got similar findings at the protein expression level; HIIT had significantly greater effect on Rac1 expression when compared to CON or to MICT. However, in our study increased expression levels were only observed in GC. Opposite results were seen on mRNA level where HIIT caused significantly *lower* expression of *Rac1* and *PAK1* in GC when compared to MICT or to the CON group. This is not unusual because it has been shown that mRNA and protein expression levels do not always correlate positively (Greenbaum et al., 2003), and it has also been suggested that mRNA levels should not even be used to predict protein levels (Fortelny et al., 2017). The differences between mRNA and protein levels on *Rac1* and *PAK1* in GC could be originated, for example, from post translational modifications, different half-lives of proteins, and error noise from experiments measuring protein and mRNA levels (Greenbaum et al., 2003). It is also known that mRNA expression is usually acutely activated, while increasing protein levels take more time. Thus, one possible explanation for the difference in direction between mRNA and protein expression could be the relatively long harvesting time (5 days) of the tissues after the exercise. Interestingly, however, significant changes in *Rac1* and *PAK1* mRNA expression were observed between training groups as well, despite the tissues not being harvested right after the intervention. To the best of our knowledge the effects of HIIT on *Rac1* and *PAK1* mRNA levels have not been studied previously. Our results suggest that HIIT might lower the mRNA expression of these genes more rapidly when compared to MICT or CON. The reason for this could be to stabilize the protein level expression (Wu and Brewer, 2012). However, more research is needed to verify these presumptions.

High-intensity interval training mainly activates fast muscle fibers and causes more mechanical stress to working muscles than MICT. Thus, this could explain higher expression levels for Rac1 as stress is greater at higher intensity, and stretch-stimulated GU is regulated by Rac1 (Sylow et al., 2015). Additionally, muscle blood flow distribution in the rat hind limb is different depending on exercise mode—swimming causes different kind of fiber recruitment patterns than treadmill exercise (Laughlin et al., 1984). It is also suggested that SOL may not be active during swimming (Armstrong and Laughlin, 1983). However, this is controversial as there are also studies showing opposite results. It is known that PGC-1α is an important regulator of skeletal muscle GU (Wende et al., 2007). Huang et al. found that moderate-intensity swimming exercise upregulated the levels of PGC-1α in SOL in rats of different ages when compared to untrained controls, although the statistical difference was only seen in the older animals (Huang et al., 2016). Slentz et al. (1992) also found that moderate-intensity swimming increased GLUT4 protein concentration and insulin-stimulated glucose transport activity in rat SOL muscle when compared to untrained controls. Finally, although the activity of SOL might be lower in swimming when compared to tread mill exercise, there are studies showing that swimming influences electromyography (EMG) activity and blood flow in the SOL muscle (Hutchison et al., 1989; Roy et al., 1991). These findings thus suggest that soleus is not completely inactive during swimming, and therefore the comparison of Rac1 and PAK1 expression levels between fast- and slow-twitch muscles in the present study is relevant. Nevertheless, some of the differences between the results of the current study and acute studies might also be explained by different exercise modes used. Moreover, the acute training effect might have already disappeared because animals in our study were not sacrificed immediately after exercise training protocols. However, even though the acute effect might have subsided, effects of HIIT especially on Rac1 expression levels were evident in GC. Importantly, this may indicate that HIIT could enhance muscle GU in GC for several days after exercise was performed.

Rac1 protein expression at baseline in mice is 40–50% higher in SOL than in GC (Sylow et al., 2013b). In line with this, we discovered that Rac1 and PAK1 mRNA and protein expression levels in CON group was higher in SOL when compared to GC, by 12, 36, 23, and 83%, respectively. These differences might be due to the heterogeneity of these muscle fibers (Woittiez et al., 1985). Additionally, slow-twitch fibers like SOL respond to insulin more effectively than fast-twitch fibers like GC (Zierath et al., 1996). In humans, GLUT4 content in slow-twitch fibers is higher as compared to fast-twitch fibers, and GLUT4 content is reduced in slow-twitch fibers in obese and type 2 diabetic subjects (Gaster et al., 2001). Rac1 and PAK1 activate GLUT4 transportation and muscle GU via exercise or insulin, so the reason for higher Rac1 expression in SOL could be related to higher GLUT4 content in slow-twitch fibers.

Further interpretations are limited by the fact that GU was not measured in GC and SOL in the present study. Relatively small sample size per group (*n* = 7-8) is also a limitation, although group sizes were based on power calculations and the ethical principles of the three Rs.

In summary, our study provides novel information on how different exercise training intensities affect Rac1 and PAK1 mRNA and protein expression in GC and SOL. HIIT caused significant *decrease* in *Rac1* and *PAK1* mRNA expression in GC compared to MICT and CON. At the protein level the results were opposite as Rac1 and PAK1 expression was higher in the HIIT group as compared to MICT and CON, although the difference in Rac1 protein expression between training groups and PAK1 or phospo-PAK1 expression between the groups were not statistically significant.

## Conclusion

The role of different exercise training intensities on Rac1 and PAK1 mRNA and protein expression levels has not been studied before. Our results suggest that high-intensity interval training (HIIT) impacts significantly especially Rac1 expression in rats but only in fast-type muscles. HIIT decreased the mRNA level of *Rac1* and *PAK1* and increased the protein level of these proteins. These findings support the notion that predicting protein expression levels from mRNA expression may not always be relevant. To conclude, HIIT appears to be important to fully activate fast muscles and especially Rac1 expression in fast skeletal muscles. Understanding how different exercise intensities affect molecular mechanisms such as Rac1 or PAK1 expression may reveal new therapeutic targets to treat patients with metabolic diseases.

## Data Availability Statement

The datasets presented in this study can be found in online repositories. The names of the repository/repositories and accession number(s) can be found below: https://doi.org/10.5281/zenodo.3950013.

## Ethics Statement

The animal study was reviewed and approved by the National Animal Experiment Board.

## Author Contributions

SL, HH, JT, MY-K, TI, TG, JR, RM, JH, KK, and IH contributed to the design of the study. MY-K, JT, and TI performed exercise interventions and sampling. SL and HH designed the laboratory analysis. RM and JR provided technical support with RPPA method, data acquisition, and analysis. SL performed the laboratory analysis, interpreted the data, and wrote the initial draft of the manuscript. SL, IH, and HH drafted the article. All authors critically revised the important content, and commented on and approved the final version of the manuscript.

## Conflict of Interest

JR and RM were employed by the company Misvik Biology. The remaining authors declare that the research was conducted in the absence of any commercial or financial relationships that could be construed as a potential conflict of interest.

## Supplementary Material

Supplementary Figure 1 Screening of Rac1, PAK1, and pPAK1. Representative Western blotting of Rac1, PAK1, and pPAK1 [phospho-PAK1 (Thr423)] in rat gastrocnemius (GC) and soleus (SOL) muscle. HSC70 was used as a loading control.
