# Supplementary material for: Effects of Different Exercise Training Protocols on Gene Expression of Rac1 and PAK1 in Healthy Rat Fast- and Slow-Type Muscles
Source: Front Physiol. 2020 Nov 19;11:584661. doi: 10.3389/fphys.2020.584661 (PMC7711069; doi:10.3389/fphys.2020.584661)
Supplement: Supplementary file 1 [file Data_Sheet_1.PDF]

Supplementary Material

Antibody screening

Materials and methods

The antibodies used in the reverse-phase protein microarray method (RPPA) were validated with Western blotting. Frozen muscle samples (GC and SOL) were homogenized in lysis buffer (1% (V/V) Triton X-100, 10 mM Tris-HCl pH 7.4, 1 mM EDTA, 10x protein inhibitor (Pierce™, Protease and Phosphatase inhibitor mini tablets, Thermo Scientific)) with F-type head tubes (Nucleospin®, Bead Tubes Type F, Macherey-Nagel) and vortexed 30 min at +4 °C. Protein concentrations were determined with the Bradford protein assay (Bio-Rad protein assay dye, cat#5000006) according to manufacturer's protocol. Gel samples were prepared in SDS sample buffer (1,7 % (m/V) SDS, 5 % (V/V) glycerol, 0,05 M Tris-HCl pH 6.8, 1,6 % (m/V) dithiothreitol (DTT), 0,002 % bromophenol blue) and heated up at 98 °C for 5 min. Proteins were separated by SDS-PAGE, and then transferred to a nitrocellulose membrane for 1 hour on ice using a wet transfer apparatus and blocked (3 % non-fat dry milk in TBS) for 30 minutes at RT in a rocker. For protein detection, antibodies against Rac1 (ab33186, Abcam) and PAK1 (PA5-18557, Thermo Fisher Scientific) were used at a dilution ratio of 1:1 000, and an antibody against phospho-PAK1 (Thr423) (PA5-12844, Thermo Fisher Scientific) at 1:500 in 3% BSA; 0.02% NaN3 in PBS and incubated overnight in tube rocker at +4 °C. Secondary antibodies anti-mouse (Horse Anti-Mouse IgG Antibody (H+L), Peroxidase PI-2000), anti-rabbit (Goat Anti-Rabbit IgG Antibody (H+L), Peroxidase PI-1000) (Vector Laboratories), anti-goat (Donkey anti-Goat IgG (H+L), HRP) and anti-rat (Goat anti-Rat IgG (H+L), HRP) (Thermo Fisher Scientific) were used at 1:10 000 dilution for 1 h at RT and detected using Pierce ECL Western Blotting Substrate (Thermo Scientific) with the Invitrogen iBright CL1000 Imaging System (Thermo Fisher Scientific). HSC70 (HSC70/HSP73 monoclonal antibody (1B5)), Enzo Life Sciences) was used as a loading control to monitor the total protein levels.

Results

Tested antibodies Rac1, phospho-PAK1 and PAK1 were expressed in *soleus* (SOL) and *gastrocnemius* (GC) muscle in male Wistar rats (**Supplement Figure 1.**)

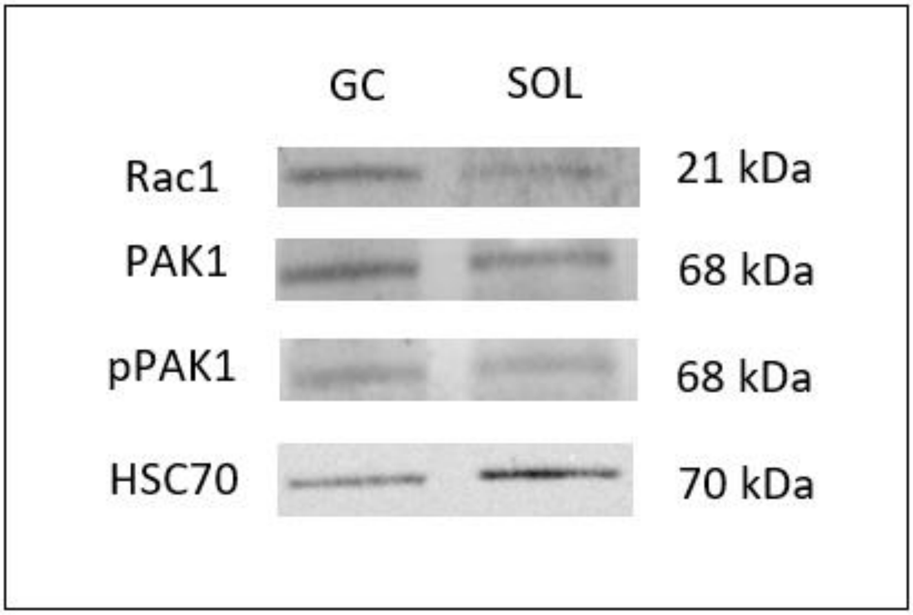

Supplement Figure 1. Screening of Rac1, PAK1 and pPAK1

Representative Western blotting of Rac1, PAK1 and pPAK1 (phospho-PAK1 (Thr423)) in rat gastrocnemius (GC) and soleus (SOL) muscle. HSC70 was used as a loading control.
