# Supplementary figures and images for: Effects of Different Exercise Training Protocols on Gene Expression of Rac1 and PAK1 in Healthy Rat Fast- and Slow-Type Muscles
Source: Front Physiol. 2020 Nov 19;11:584661. doi: 10.3389/fphys.2020.584661 (PMC7711069; doi:10.3389/fphys.2020.584661)

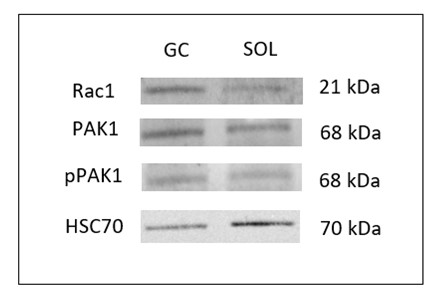

Supplement: Supplementary Figure 1 — Screening of Rac1, PAK1, and pPAK1. Representative Western blotting of Rac1, PAK1, and pPAK1 [phospho-PAK1 (Thr423)] in rat gastrocnemius (GC) and soleus (SOL) muscle. HSC70 was used as a loading control. [file Image_1.JPEG]
